# Remote Monitoring Is Only as Safe as Its Response Pathway: Why Pediatric CIED Reports Should Include Alert Burden, False Positives, and Response‐Time Standards

**DOI:** 10.1002/ccr3.72887

**Published:** 2026-06-08

**Authors:** Muhammad Abdullah Awan, Hammad‐ud‐din Raza

**Affiliations:** ^1^ Wah Medical College National University of Medical Sciences Rawalpindi Pakistan; ^2^ Wah Medical College Wah Pakistan

**Keywords:** alert fatigue, cardiac implantable electronic devices, pacemaker, pediatric arrhythmia, remote monitoring, response time

## Abstract

Daily remote monitoring of pediatric cardiac implantable electronic devices is clinically valuable, but its reproducibility depends on more than arrhythmia detection. Future reports should include alert burden, false‐positive rates, alert‐to‐review intervals, family‐contact timelines, and escalation pathways to clarify whether remote monitoring systems are operationally safe and scalable.

Dear Editor,

We read with great interest the case series by Wieniawski et al., titled “Daily Remote Monitoring of Pediatric Patients With Cardiac Implantable Electronic Devices: Early Arrhythmia Detection and Timely Clinical Intervention,” published in Clinical Case Reports [1]. The authors present three pediatric patients with cardiac implantable electronic devices (CIEDs) in whom daily remote monitoring (RM) detected clinically important arrhythmias, including atrial fibrillation degenerating into ventricular fibrillation in an adolescent with intellectual disability, asymptomatic atrial flutter in a 6‐year‐old with repaired tetralogy of Fallot, and recurrent atrial flutter in a 2‐year‐old child [1]. The report is valuable because it highlights a central pediatric problem: children may be unable to recognize, report, or communicate clinically significant arrhythmias.

However, one point deserves stronger emphasis: RM is only as safe as the clinical response pathway that follows the alert. The authors appropriately describe red and yellow alert categories, device‐specific thresholds, and automatic email or text‐message notifications to the assigned clinician [1]. Yet the report does not quantify the total alert burden, the number of non‐actionable alerts, false‐positive transmissions, time from alert generation to physician review, time from review to family contact, or time from contact to clinical intervention. Without these operational metrics, it is difficult to judge how reproducible the model would be in centers with different staffing structures, after‐hours coverage, or patient volumes.

This distinction is clinically important because pediatric RM may generate a high signal‐to‐noise challenge. Wieniawski et al. note that sinus tachycardia caused by crying, agitation, or febrile illness may produce false‐positive yellow alerts, and that alert evaluation and response decisions remain the responsibility of a single designated physician [1]. This is a practical and honest observation, but it also raises a scalability question. A single‐physician review model may function well in a small descriptive series, but larger pediatric CIED programs require defined triage rules, backup review coverage, response‐time expectations, and documentation of alert actionability to prevent clinician overload or delayed response to true emergencies.

For future reports, we suggest that pediatric RM outcomes should be reported using a minimum operational dataset. This could include the number of total transmissions, red alerts, yellow alerts, arrhythmia‐confirmed alerts, false‐positive alerts, device‐related alerts, connectivity failures, median alert‐to‐review time, median alert‐to‐family‐contact time, and median alert‐to‐intervention time. Such reporting would help distinguish the diagnostic performance of the device from the performance of the monitoring system. In pediatric practice, the second component is especially important because parents are often the point of contact and clinical decisions may depend on rapid communication with caregivers.

The three cases also show why response pathways should be disease‐ and age‐specific. In Case 1, the patient and family were unaware that an ICD shock had occurred, making RM essential for identifying a life‐threatening event [1]. In Cases 2 and 3, pre‐admission recognition of atrial flutter allowed the team to prepare for electrical cardioversion and avoid additional diagnostic delay [1]. These are strong examples of clinical utility. Nevertheless, the report would be even more instructive if it clarified the exact time intervals between RM alert delivery, clinician recognition, family notification, hospital arrival, and cardioversion or other intervention. These intervals are the practical measures that determine whether RM truly shortens care pathways.

Another issue is parental communication. The authors correctly emphasize that parents are central to pediatric RM implementation [1]. We suggest that future case reports specify whether families receive written alert instructions, whether red and yellow alerts are explained at enrollment, what caregivers are told to do when contacted after an alert, and how loss of RM connectivity is handled. In children with complex congenital heart disease, neurodevelopmental impairment, or prior sternotomy and epicardial systems, the family‐facing part of the response pathway may be as important as the technical alert itself.

Importantly, our comments do not weaken the authors' conclusion that daily RM can be useful in pediatric CIED follow‐up. Rather, they support the next step proposed by the authors: prospective registry‐based evaluation [1]. Such a registry should capture not only arrhythmia detection and hospitalization outcomes, but also alert workload, clinician response times, false‐positive rates, caregiver contact success, and staffing sustainability. These data would make pediatric RM more comparable across centers and would help define safe minimum standards for daily monitoring programs.

Overall, Wieniawski et al. provide an educational and clinically relevant case series demonstrating that daily RM can identify silent but significant arrhythmias in children with CIEDs [1]. Its broader lesson is that detection is only the first half of remote care. For RM to become a reproducible standard in pediatric practice, future reports should describe the complete response architecture behind the alert: who reviews it, how quickly it is reviewed, how often it is false‐positive, how families are reached, and when escalation occurs.

This case series convincingly illustrates the diagnostic value of daily RM in pediatric CIED patients who are asymptomatic or unable to self‐report symptoms. However, future pediatric RM reports should include alert burden, false‐positive frequency, response‐time metrics, caregiver communication pathways, and escalation criteria. These details would transform case‐based evidence from proof of detection into a practical model for safe and scalable remote pediatric cardiac care.

## Author Contributions

**Muhammad Abdullah Awan:** conceptualization, formal analysis, project administration, writing – original draft, writing – review and editing. **Hammad‐ud‐din Raza:** data curation, writing – original draft, writing – review and editing.

## Funding

The authors have nothing to report.

## Ethics Statement

The authors have nothing to report.

## Consent

The authors have nothing to report.

## Conflicts of Interest

The authors declare no conflicts of interest.

## Data Availability

The authors have nothing to report.
